# Exploring the influence of DNA methylation and single nucleotide polymorphisms of the *Myostatin* gene on growth traits in the hybrid grouper (*Epinephelus fuscoguttatus* (female) × *Epinephelus polyphekadion* (male))

**DOI:** 10.3389/fgene.2023.1277647

**Published:** 2024-01-08

**Authors:** Liu Cao, Jun Ma, Pan Chen, Xingrong Hou, Ning Yang, Yan Lu, Hai Huang

**Affiliations:** ^1^ Yazhou Bay Innovation Institute, Sanya, China; ^2^ Hainan Key Laboratory for Conservation and Utilization of Tropical Marine Fishery Resources, Sanya, China; ^3^ Key Laboratory of Utilization and Conservation for Tropical Marine Bioresources of Ministry of Education, Sanya, China; ^4^ College of Fisheries and Life Sciences, Hainan Tropical Ocean University, Sanya, China

**Keywords:** growth trait, *mstn* gene, SNP, DNA methylation, grouper

## Abstract

Investigations into the correlation between growth characteristics and DNA methylation levels, along with genetic variations, can provide fundamental insights to enhance growth performance in groupers. The *Myostatin* (*mstn*) gene plays a vital role in regulating skeletal muscle development and growth. This study scrutinized the DNA methylation levels of the *mstn* gene across hybrid groupers (*E. fuscoguttatus* (♀) × *E. polyphekadion* (♂)) and their parental species, to evaluate its impact on growth attributes in grouper fish. The nucleotide sequence of the *mstn* gene was directly sequenced in the hybrid grouper, exhibiting different growth performance to identify the single nucleotide polymorphisms (SNPs) of the *mstn* gene and explore their correlation with growth characteristics. The findings revealed no significant differences in global DNA methylation levels within muscle tissue among the hybrid grouper and parents. However, significant differences in DNA methylation sites were discovered between the hybrid grouper and *E. polyphekadion* at sites 824 and 1521 (located at exon 2 and intron 2, respectively), and between *E. fuscoguttatus* and *E. polyphekadion* at site 1521. These variations could potentially influence the mRNA expression of the *mstn* gene. The study also identified that SNP g.1003 T > C in exon 2 of the *mstn* gene was significantly associated with various growth traits including body weight, total length, body length, head length, caudal peduncle height, and body height (*p* < 0.01). Specimens with the TT genotype at site 1003 demonstrated superior growth performance compared to those with the TC genotype. Furthermore, microstructural analyses of muscle tissue showed that the average area and diameter of muscle fibers in TT genotype individuals were significantly greater than those in TC genotype individuals. Therefore, this research provides robust evidence linking the DNA methylation level and polymorphisms of the *mstn* gene with growth traits, which could be beneficial for grouper breeding programs.

## 1 Introduction

The grouper is a highly prized marine fish, thanks to its excellent nutritional content and limited availability in natural waters. It is a leading aquaculture species in the southern coastal regions of China. In 2021, grouper aquaculture production exceeded 200,000 tons (FAO, 2022). Hainan presently stands as China’s major grouper producer, with the production of grouper reaching 70,178 tons in 2021, according to the China Fishery Statistics Yearbook. This figure closely trails the production from Guangdong. The primary aquaculture species in Hainan is the hybrid grouper (*Epinephelus fuscoguttatus* (♀) × *Epinephelus polyphekadion* (♂)) (Cao et al., 2022). Given the direct impact of growth traits on farming benefits, understanding the molecular mechanisms driving growth in hybrid groupers is imperative.

DNA methylation, a primary mechanism that regulates gene expression (Ding et al., 2012; Si et al., 2016; Li et al., 2017), can induce phenotypic changes (Jaenisch and Bird, 2003; Angers et al., 2010). An increasing body of evidence underscores the influence of DNA methylation on growth in animals (Zhong et al., 2014; Yang and Li, 2021). Single nucleotide polymorphisms (SNPs) are another crucial resource for genetic association studies (Zhang et al., 2015; Liu et al., 2016). A significant correlation between SNP markers and traits suggests a possible association between these markers and certain characteristics (Doerge, 2002). The discovery of growth-related SNP markers in several fish species, including *Danio rerio*, *Oncorhynchus mykiss*, *Hemibagrus wyckioides*, and *Epinephelus coioides* (Salem et al., 2012; Ulloa et al., 2015; Guo et al., 2016; Zhou et al., 2021), implies that selective breeding based on these markers holds promise for enhancing growth traits.

*Myostatin* (*mstn*, also known as GDF-8), a member of the transforming growth factor-β superfamily, expresses in various fish tissues including gills, brain, ovaries, and skeletal muscle (Rodgers et al., 2001; Ko et al., 2007). The *mstn* gene acts as a negative regulator of muscle growth in skeletal muscle by suppressing the hypertrophy and hyperplasia of muscle cells (McPherron et al., 1997; Lee and McPherron, 1999). This mechanism has been identified in several aquatic species, such as *O. mykiss* (Nazari et al., 2016), *Litopenaeus vannamei* (Qian et al., 2013), *Argopecten irradians* (Meng et al., 2017), *Echinoidea* (Lapraz et al., 2006), and *Paralichthys olivaceus* (Zhong et al., 2008). Numerous studies have explored the relationships between various nucleotide polymorphisms of the *mstn* gene and growth traits in commercial species. These investigations provide valuable genetic markers for the genetic improvement of aquatic species (Sun et al., 2012; Peñaloza et al., 2013; Meng et al., 2017). Currently, the sequence variants and methylation status of the *mstn* gene in hybrid grouper remain under-explored. Therefore, this study aims to examine the correlation between growth traits and the methylation status, expression pattern, and polymorphisms of the *mstn* gene in grouper, in an effort to unearth potential markers for assisted selection.

## 2 Materials and methods

### 2.1 Experimental animals and samples collection

Specimens of *E*. *fuscoguttatus*, *E*. *polyphekadion*, and the hybrid grouper were procured and reared at the Hainan Chenhai Aquatic Co. Ltd. In Hainan Province, China. To ensure optimal conditions, the health of the fish and environmental parameters were closely monitored on a daily basis. All experimental procedures strictly adhered to the guidelines stipulated by Administration of Affairs Concerning Animal Experimentation of China.

### 2.2 DNA methylation analysis

Muscle tissues were utilized for genomic DNA extraction in *E. fuscoguttatus* (five individuals), *E. polyphekadion* (five individuals), and the hybrid grouper (six individuals). Genomic DNA (1 μg) was processed using the ZYMO EZ DNA Methylation-Gold Kit (Zymo Research, Irvine, CA, United States), and one-tenth of the elution products were employed as templates. PCR primers were developed using the MethPrimer software package (www.urogene.org/methprimer/index.html), which was guided by the nucleotide sequence of the *mstn* gene (Cao et al., 2023) (refer to Supplementary Figure S1). PCR amplification was performed for 35 cycles, with an annealing duration of 30 s at 50–60°C, using the KAPA HiFi HotStart Uracil + ReadyMix PCR Kit (Kapa Biosystems, Wilmington, MA, USA). For each sample, bisulfite sequencing PCR (BSP) products (∼200 bp in length) were evenly pooled, 5′-phosphorylated, 3′-dA-tailed, and ligated to a barcoded adapter using T4 DNA ligase (NEB). Barcoded libraries from all samples were pooled uniformly and utilized for pair-end sequencing using the Illumina platform (Gao et al., 2015). The raw reads were first processed to remove adapters and filter out low-quality sequences using Trimmomatic-0.36, yielding clear reads. These clean reads were then aligned to the reference sequence (*mstn* gene) using BSMAP (Version 2.73). The methylation levels of individual cytosines were calculated as the ratio of the total number of methylated CpG cytosines to the number of sequenced clones. The global DNA methylation level was calculated as the average methylation levels of all CpG sites in each sample. For the methylation level of each CpG site, global DNA methylation level of *mstn* gene, comparisons between two groups were conducted using a two-tailed Fisher’s Exact Test with *p* < 0.05 indicating a statistically significant difference (Gao et al., 2014).

### 2.3 SNP genotyping and statistical analysis

The hybrid grouper was reared in seawater tank (4 m × 4 m) under identical conditions and for the same duration. After 195 days of culture, their body weight and morphological indicators were measured. Individuals with slow-growth and fast-growth were selected and divided into two groups (slow-growth group and fast-growth group) according to the value of body weight. The mean value of body weight in slow-growth group and fast-growth group was 72.67 g and 196.67 g, respectively. In these two groups, individuals were excluded when their body weight was deviate significantly from the mean body weight. Finally, 51 specimens (24 individuals derived from the slow-growth group, 27 individuals derived from the fast-growth group) were collected to explore the potential influence of SNPs in the *mstn* gene on the growth traits of hybrid groupers. Genomic DNA was extracted from muscle tissues using the Animal Genomic DNA Extraction Kit as per the manufacturer’s instructions (Sangon Biotech, Shanghai). The DNA samples’ concentration and quality were assessed through 1% agarose gel electrophoresis. Three pairs of PCR primers, designed using Primer 5.0 and synthesized by Sangon Biotech (Guangzhou, China), were employed to clone and identify SNP sites in the *mstn* gene (Supplementary Table S1). Genotyping was carried out through direct sequencing (Sangon Biotech, Guangzhou) using an Applied Biosystems ABI 3730 L Genetic Analyzer (Applied Biosystems group, US), and the resulting sequences were analyzed via Chromas.

Genotype frequencies and gene frequencies at SNP sites were computed according to the following formulas (Lyu et al., 2020): PAA = NAA/N (PAA: frequency of AA genotype; NAA: number of AA genotype specimens; N: total number of hybrid groupers); PA=(2NAA + NAG)/2N, PG=(2NGG + NAG)/2N (PA, PG: frequencies of A and G alleles; NAA, NAG, and NGG: number of individuals with AA, AG, and GG genotypes; N: total number of hybrid groupers). The polymorphic information content (PIC) and expected heterozygosity (He) were computed using Curves 3.0.7 software, Linkage disequilibrium (LD) was calculated with Haploview 4.2 software. Ultimately, only two SNPs (minor allele frequency >0.01, *p*-value for Hardy-Weinberg equilibrium test >0.05, PIC >0.2) were selected for further analysis.

Pearson correlation coefficients were determined using SPSS 17.0 to elucidate correlations between various growth traits. The associations between the SNP site and growth traits were analyzed using the Generalized Linear Model (GLM) procedure in the R package.

### 2.4 Quantitative real-time PCR analysis

The relative expression levels of *mstn* mRNA were analyzed using muscle cDNA sourced from *E. fuscoguttatus*, *E. polyphekadion*, and the hybrid grouper. To facilitate this, specific primers were designed using the Primer 5.0 software (Supplementary Table S1). Quantitative PCR (qPCR) was conducted on a Step One Plus™ Real-Time PCR System (Applied Biosystems) utilizing the ChamQ SYBR qPCR Master Mix (Vazyme), with protocols adhering to manufacturer guidelines. The reaction mix, with a total volume of 10 μL, was composed of 5 µL of 2 × qPCR mix, 0.25 µL of both forward and reverse primers, 2 µL of DNA template, and 2.5 µL of RNase-free water. The qPCR reaction was initiated with a pre-denaturation step at 95°C for 30 s, followed by 40 cycles of denaturation at 95°C for 10 s and annealing/extension at 60°C for 30 s. The *β-actin* gene was used as a reference gene for normalization. Each sample was run in triplicate using the same primers. The relative gene expression was determined using the 2^−ΔΔCT^ method (Livak and Schmittgen, 2013).

### 2.5 Histological analysis

White muscle fiber samples were obtained from the dorsal region of hybrid groupers, each having different genotypes at site 1,003 (three individuals each from TT and TC genotypes). All samples were dissected into pieces of 0.2 cm³ and subjected to sequential dehydration through a range of alcohol concentrations. Subsequently, the tissues were processed with an alcohol:xylene (1:1) solution, followed by 100% xylene, and then embedded in paraffin wax. The paraffin-embedded samples were sliced into serial sections for Hematoxylin and Eosin (HE) staining. Three fields of view were chosen for each sample to measure the muscle fiber cross-sectional area and diameter using Image-Pro Plus 6.0 software. Statistical analyses of the data were carried out using the Analysis of Variance (ANOVA) test in SPSS 17.0 software.

## 3 Results

### 3.1 Global DNA methylation levels of *mstn* gene

**FIGURE 1:**
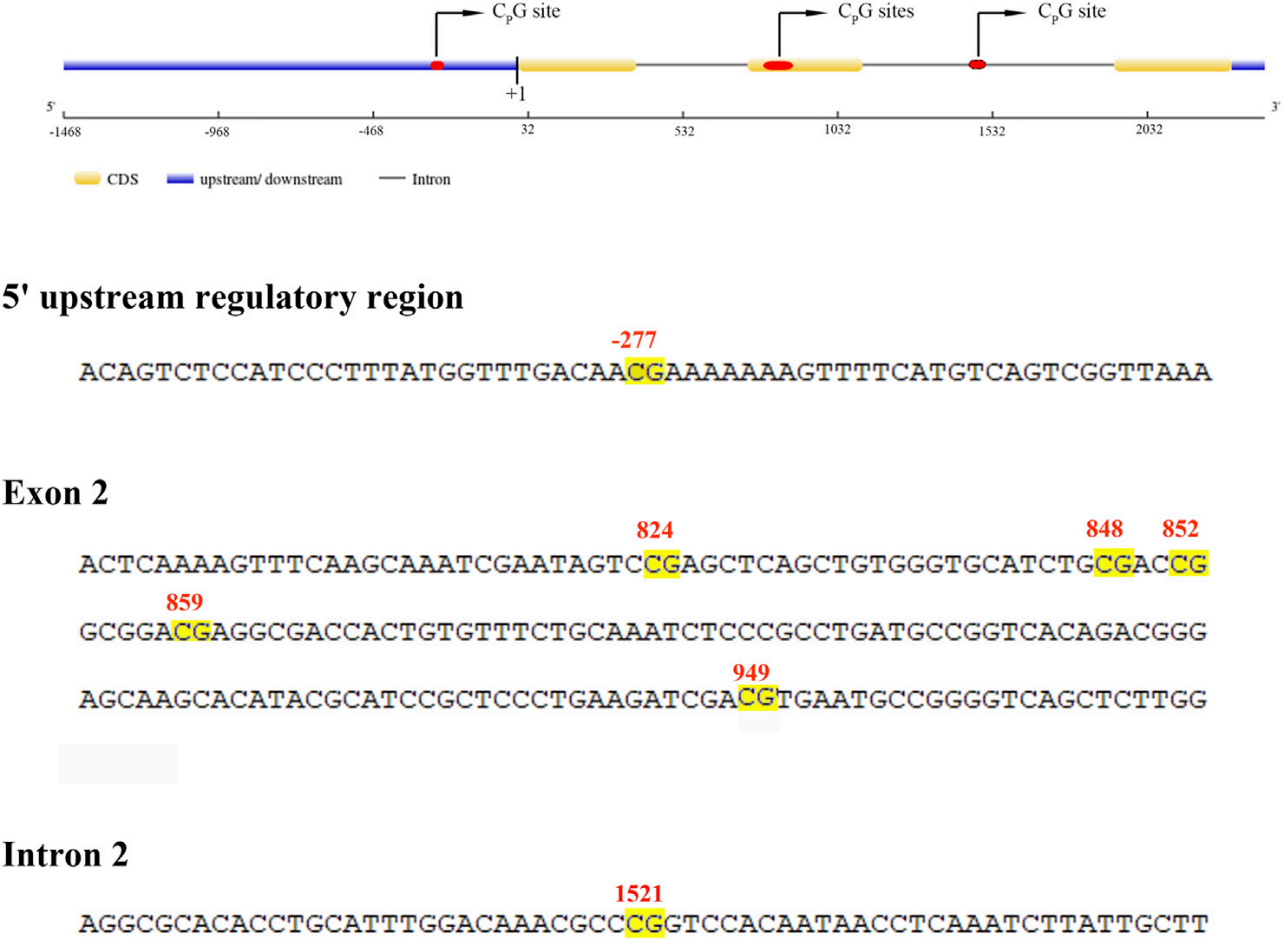
Schematic organization and partial sequence of *mstn* gene. The location of the CpG sites are highlighted by a yellow background.

**FIGURE 2:**
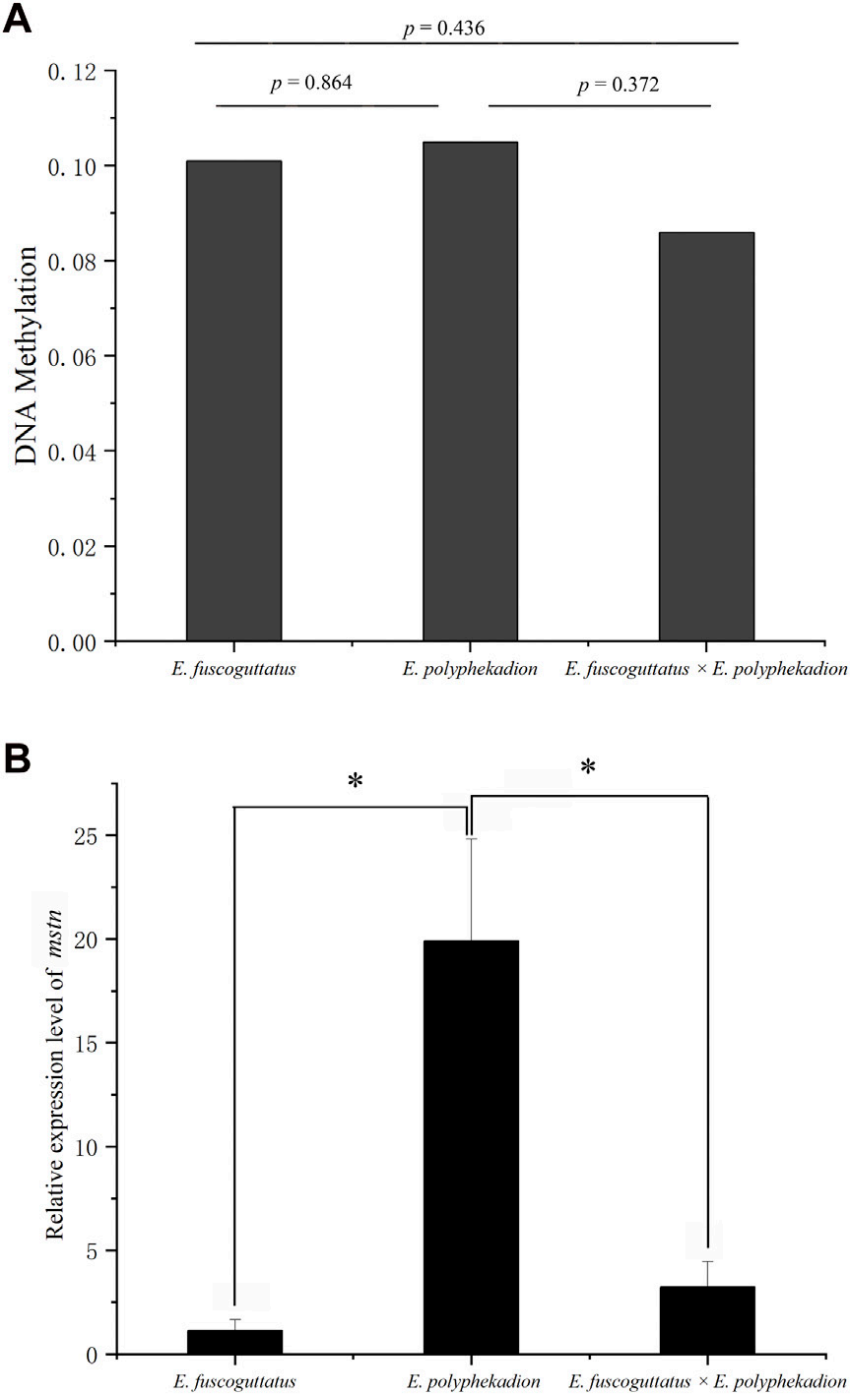
**(A)** The global of DNA methylation levels of *mstn* gene in *Epinephelus fuscoguttatus*, *Epinephelus polyphekadion* and the hybrid grouper; **(B)** Expression levels of *mstn* gene in *Epinephelus fuscoguttatus*, *Epinephelus polyphekadion* and the hybrid grouper. * indicates there is a significant difference between two groups.

The entire length of the *mstn* gene, including a 1,468 base pair (bp) upstream fragment adjacent to the transcription start site (TSS), three exons, and two introns (Figure 1), was evaluated to assess the global level of DNA methylation across the hybrid grouper and its parental species. The data revealed that the comprehensive DNA methylation levels of the *mstn* gene were relatively low in all groupers, with no significant differences (*p* > 0.05) observed between the hybrid grouper (0.0855), *E. fuscoguttatus* (0.1009), and *E. polyphekadion* (0.1047) (Figure 2A). This suggests that methylation does not vary significantly based on species. However, there were notable differences in the mRNA expression levels of the *mstn* gene between *E. polyphekadion*, *E. fuscoguttatus*, and the hybrid grouper (Figure 2B).

### 3.2 Single CpG site methylation levels of *mstn* gene

**FIGURE 3:**
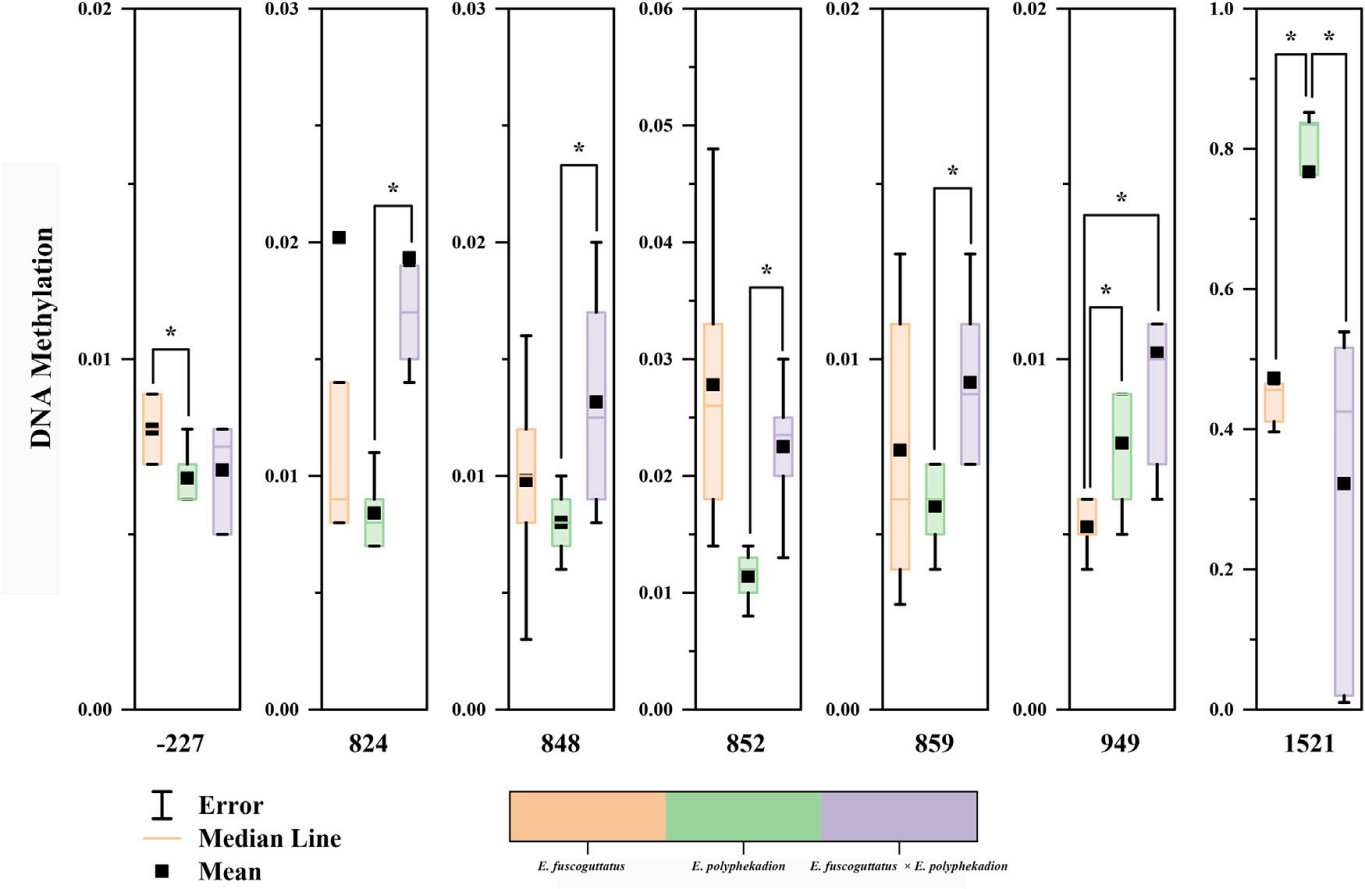
The site-specific DNA methylation levels of *mstn* gene in *Epinephelus fuscoguttatus*, *Epinephelus polyphekadion* and the hybrid grouper. *X*-axis represents the position of the cytosine in the CpG dinucleotide context relative to the transcription start site. * indicates there is a significant difference between two groups.

Additionally, the CpG sites in the 5′upstream regulatory region and the exon and intron of the *mstn* gene were examined to discern differences in methylation levels between the hybrid grouper and its parent species. Notable regional differences in *mstn* gene DNA methylation were detected in the 5′upstream regulatory region (position −227), exon 2 (positions 824, 848, 852, 859, and 949), and intron 2 (position 1,521) (Figure 3). Significant methylation differences between *E. fuscoguttatus* and *E. polyphekadion* were found at positions −227, 949, and 1,521. At site −227, the DNA methylation level in *E. polyphekadion* (0.0066) was lower than that in *E. fuscoguttatus* (0.0080). Conversely, the DNA methylation patterns at sites 949 and 1,521 showed an inverse relationship between *E. fuscoguttatus* (0.0052 and 0.4730, respectively) and *E. polyphekadion* (0.0076 and 0.7672, respectively).

The DNA methylation levels exhibited significant differences between the hybrid grouper and its male parent at positions 824, 848, 852, 859, and 1,521. Generally, the hybrid grouper demonstrated significantly higher DNA methylation levels at all these positions, compared to its male parent (except at position 1,521). The hybrid grouper and the female parent only displayed a significant difference at one CpG site (position 949), where the DNA methylation level in the hybrid grouper (0.0102) exceeded that of the female parent (0.0052).

### 3.3 Correlation between DNA methylation levels and gene expression

**FIGURE 4:**
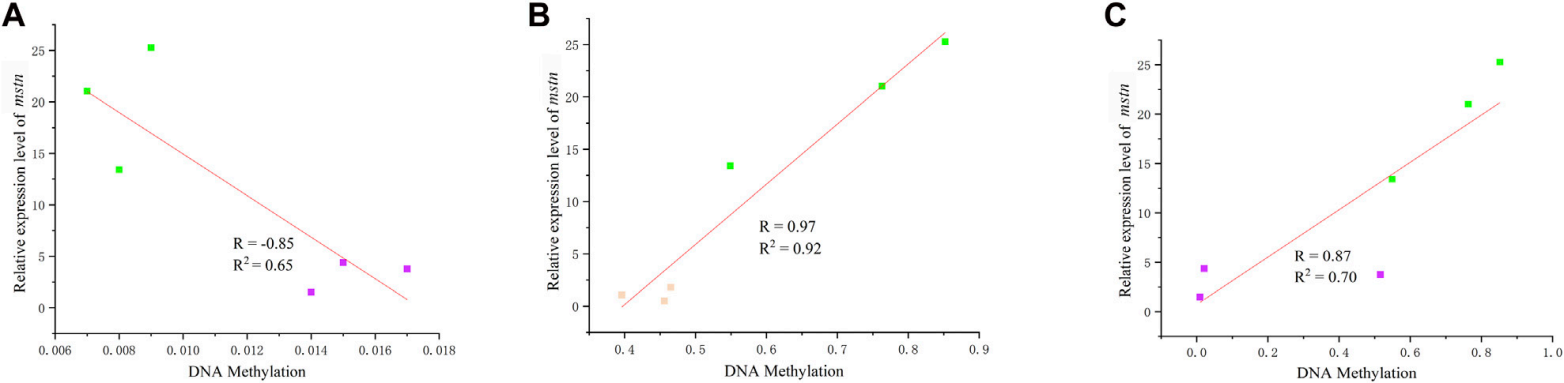
The correlation between DNA methylation and gene mRNA expression. **(A)** Correlation between DNA methylation at site 824 and gene mRNA expression in *Epinephelus polyphekadion* and the hybrid grouper. **(B)** Correlation between DNA methylation at site 1,521 and gene mRNA expression in *Epinephelus polyphekadion* and *Epinephelus fuscoguttatus.* **(C)** Correlation between DNA methylation at site 1,521 and gene mRNA expression in *Epinephelus polyphekadion* and the hybrid grouper. Orange represents *Epinephelus fuscoguttatus,* green represents *Epinephelus polyphekadion* and purple represents the hybrid grouper.

The relative expression level results revealed no significant change between the hybrid grouper and *E. fuscoguttatus*, suggesting that the DNA methylation level at site 949 does not influence relative expression. However, potential impacts from the methylation of other CpG sites are considered herein. The methylation levels at CpG sites 824 and 1,521 exhibited a notable correlation with the expression of the *mstn* gene (Figure 4). Figure 4A illustrates the association between gene expression and the methylation level at site 824. A negative correlation was observed between *mstn* mRNA levels and DNA methylation (R = −0.85, *p* < 0.05) when comparing the hybrid grouper and *E. polyphekadion*. Conversely, the DNA methylation level of *mstn* was positively correlated with gene expression at site 1,521 (Figures 4B, C). The correlation coefficients between CpG methylation and gene expression were 0.97 and 0.87 when comparing *E. fuscoguttatus* and *E. polyphekadion*, and the hybrid grouper and *E. polyphekadion*, respectively.

### 3.4 Single nucleotide polymorphisms in the *mstn* gene

To identify SNP sites associated with growth traits, this study analyzed loci within or near the coding region of the *mstn* gene in hybrid grouper. SNPs that did not comply with the Hardy-Weinberg equilibrium were excluded from this investigation. The mean values and ranges for each trait in the hybrid grouper are presented in Table 1. Pearson correlation coefficients were computed to reveal relationships among various traits (Table 2). Significant differences were observed in the Pearson correlation coefficients between two distinct traits, indicating a strong positive correlation among all traits. Notably, the coefficients between total length and both body weight and body length were extremely close to 1, suggesting a high correlation between these attributes. Details regarding the SNPs of the *mstn* gene are provided in Table 3. Two SNPs (g.818 A>G, g.1003 T>C) (Supplementary Figure S2) adhered to the Hardy-Weinberg equilibrium (*p* > 0.05). These two SNPs exhibited polymorphism with minor allele frequencies >1% and a high level of heterozygosity (PIC >0.5).

**TABLE 1:** Means ± SD and the range for each trait in the hybrid grouper.

Traits	Mean ± SD	Range	
		Minimum	Maximum
Body weight (g)	139.41 ± 63.91	60	240
Total length (cm)	19.44 ± 3.21	15	24.2
Body length (cm)	16.79 ± 2.73	12.7	21.4
Head length (cm)	6.03 ± 1.02	4.4	8.2
Caudal peduncle length (cm)	2.53 ± 0.41	1.8	3.2
Body height (cm)	5.48 ± 1.25	3.63	7.8
Caudal peduncle height (cm)	1.91 ± 0.36	1.3	2.6

**TABLE 2:** Pearson correlation coefficients between each trait in the hybrid grouper.

	Body weight	Total length	Body length	Head length	Caudal peduncle length	Body height	Caudal peduncle height
Body weight	1	0.983^*^	0.961^*^	0.925^*^	0.788^*^	0.955^*^	0.934^*^
Total length			0.983^*^	0.953^*^	0.796^*^	0.946^*^	0.941^*^
Body length				0.932^*^	0.807^*^	0.923^*^	0.927^*^
Head length					0.705^*^	0.898^*^	0.899^*^
Caudal peduncle length						0.763^*^	0.752^*^
Body height							0.956^*^

Note:^*^ there is a significant difference (*p* < 0.01).

**TABLE 3:** Genotypic and allelic frequencies of SNPs in *mstn* and Hardy-Weinberg equilibrium test.

SNP	Location	N	Genotypic frequency		Allelic frequency			He	PIC
			Homozygous	Heterozygous	Major	Minor	HWpval		
g.818	Exon 2	51	0.529 (27)	0.471 (24)	0.765	0.235	0.0563	0.987	0.965
g.1003	Exon 2	51	0.745 (38)	0.255 (13)	0.873	0.127	0.8343	0.987	0.949

Note: HWpval: *p*-value for Hardy-Weinberg equilibrium test; He: expected heterozygosity; PIC, polymorphic information content.

### 3.5 Association between SNPs of *mstn* gene and growth traits

We further conducted experiments to ascertain potential impacts of SNPs within muscle DNA. Notably, we found that SNP g.1003 showed a significant association with several growth attributes, including body weight, total length, body length, head length, body height, and caudal peduncle height (*p* < 0.01; refer to Table 4). Individuals possessing the TT genotype for SNP g.1003 demonstrated superior growth performance compared to those with the TC genotype (*p* < 0.01).

**TABLE 4:** Least squares means ± SEM among the SNP of *mstn* and each traits.

SNP	g.1003	
	(TT)	(TC)
Body weight (g)	161.84 ± 59.07^a^	73.85 ± 7.38^b^
Total length (cm)	20.53 ± 3.00^a^	16.25 ± 0.64^b^
Body length (cm)	17.68 ± 2.59^a^	14.19 ± 0.73^b^
Head length (cm)	6.33 ± 0.98^a^	5.13 ± 0.39^b^
Caudal peduncle length (cm)	2.64 ± 0.39	2.21 ± 0.24
Caudal peduncle height (cm)	2.02 ± 0.34^a^	1.57 ± 0.14^b^
Body height (cm)	5.91 ± 1.16^a^	4.22 ± 0.33^b^

Note: Among different genotypes within each SNP, in the same line, values with different lowercase superscripts denote significant differences (*p* < 0.01).

### 3.6 Histological comparison of hybrid groupers with different growth rate

**FIGURE 5:**
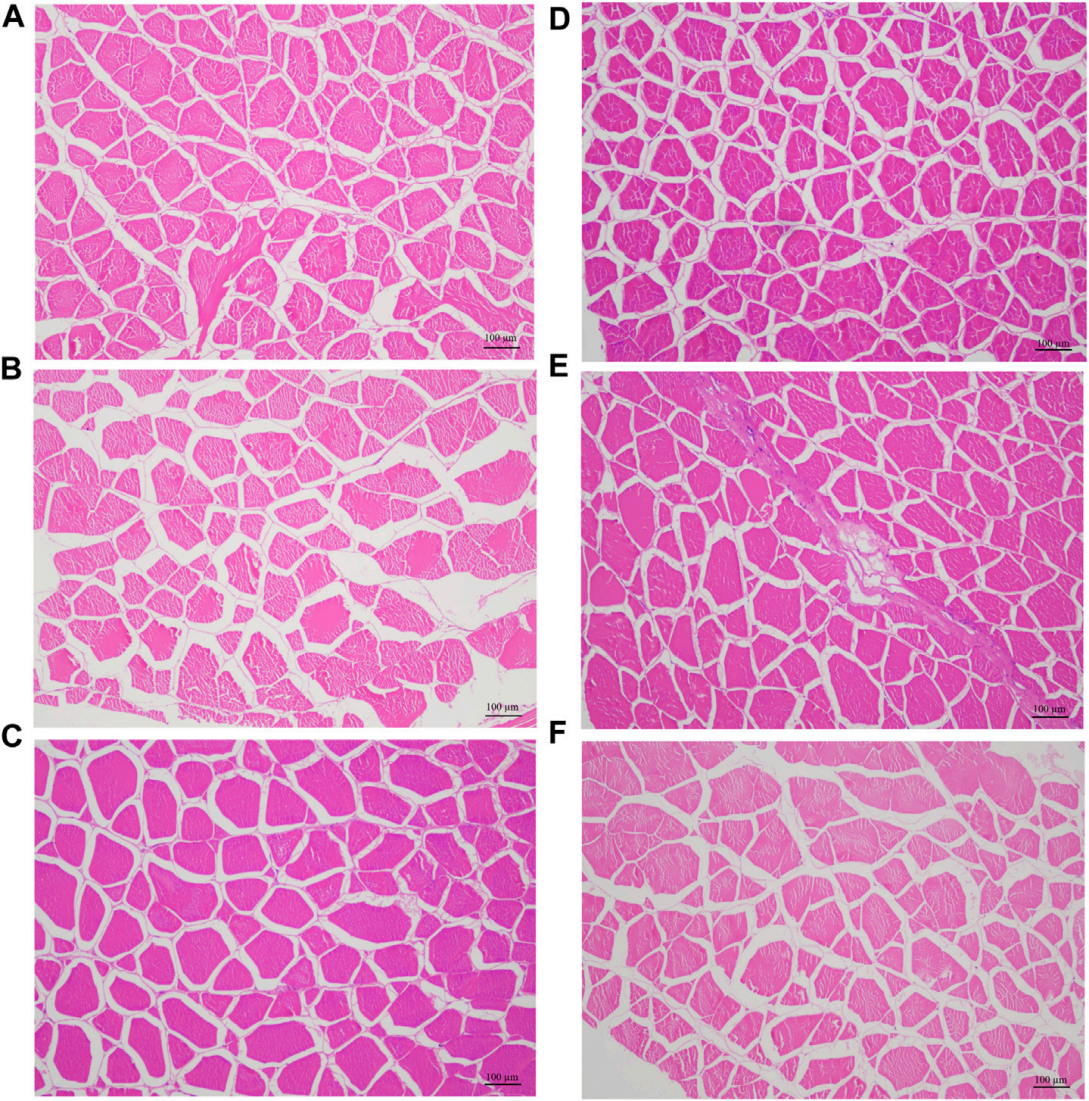
Histological characteristic of myofiber in six hybrid grouper, three with TT genotype **(A–C)** and three with TC genotype **(D–F)**.

In accordance with the genotyping of the SNP g.1003 in *mstn* gene, we selected hybrid groupers with TT and TC genotypes, three individuals each for microscopic examination (Figure 5; Table 5). As indicated in Table 5, the average cross-sectional area and diameter of muscle fibers in the hybrid groupers with the TT genotype (1,969.03 ± 8.07 µm^2^, 25.02 ± 0.91 µm, respectively) were significantly greater than those of the groupers with the TC genotype (1,330.34 ± 23.87 µm^2^, 21.43 ± 0.49 µm) (*p* < 0.01). Hematoxylin and Eosin (HE) staining results showed that muscle fibers in individuals with the TT genotype were generally thicker than those in individuals with the TC genotype.

**TABLE 5:** The measurement of the muscle fiber cross-sectional area and diameter in the hybrid grouper with different growth rate.

Group	Area (µm^2^)	Average ± standard error	Diameter (µm)	Average ± standard error
TT genotype	1,959.13	1,969.03 ± 8.07^a^	26.20	25.02 ± 0.91^a^
	1,969.05		24.86	
	1,978.90		23.99	
TC genotype	1,355.88	1,330.34 ± 23.87^b^	20.74	21.43 ± 0.49^b^
	1,336.68		21.68	
	1,298.46		21.86	

Note: In the same column, values with different lowercase superscripts denote significant differences (*p* < 0.01).

## 4 Discussion

Hybridization can induce alterations in genes, epigenetics, and phenotypic traits (Baack and Rieseberg, 2007). Epigenetic factors, which are chemically stable, influence gene expression, thereby modifying the phenotype during animal development (Ngô et al., 1996; Santos et al., 2005; Potok et al., 2013; Zhang et al., 2019). The *mstn* gene, acting as a powerful inhibitor of muscle growth, predominantly expresses in skeletal muscle (Rios et al., 2002). Several studies have documented a negative correlation between the methylation status of the *mstn* gene and its expression (Liu et al., 2010; Yao et al., 2017). In this study, the global methylation levels of the *mstn* gene were generally low, with no significant differences observed between the hybrid grouper, *E. fuscoguttatus*, and *E. polyphekadion*. However, significant variations in the *mstn* gene’s expression level were evident between *E. fuscoguttatus* and *E. polyphekadion*, and between the hybrid grouper and *E. polyphekadion*. Mark et al. (1998) revealed that methylation of around 60% of a 5-CpG island could completely inhibit gene expression, while a lower degree of methylation only reduces gene transcription and expression (Mark et al., 1998). Therefore, we deduce that the main role of *mstn* gene methylation in this study is to decrease gene transcription and expression.

A comparative analysis of each CpG site’s methylation status unveiled significant differences in methylation levels at three CpG sites between *E. fuscoguttatus* and *E. polyphekadion*. A correlation between methylation level and gene expression was observed only at site 1,521 (R = 0.97). This positive correlation was also obvious between the hybrid grouper and *E. polyphekadion* (R = 0.87). Typically, high DNA methylation patterns indicate low mRNA expression. A significant positive correlation between gene expression and DNA methylation has been observed in animals and plants, such as *Mus musculus* (Kobayashi et al., 2012), *Sus scrofa* (Xiao et al., 2014), *sacred lotus* (Li et al., 2021). DNA methylation on promoter or gene body can have complex regulatory roles, in some cases inhibiting gene expression and in others inducing expression (Lang et al., 2017; Huang et al., 2019). Because DNA methylation might cause alterations in chromatin structure, thereby modifying the interactions between DNA and activating or repressing transcription factors (or complexes) (Santos et al., 2005). Consequently, methylation might enhance transcription by preventing the binding of negative regulatory factors (Lai et al., 2010), or increasing the binding of positive regulatory factors (Lorincz et al., 2004; Kulis et al., 2012). In addition, methylation status at site 824 displayed a negative correlation with gene expression between the hybrid grouper and *E. polyphekadion* (R = −0.85). DNA methylation in exons might collaboratively regulate splicing (Schwartz et al., 2009; Schwartz and Ast, 2010). Based on these foundings in this study, it is speculated that the influence of DNA methylation is not determined by single CpG site. Further molecular genetic studies are needed to validate this hypothesis.

The *mstn* gene has been extensively investigated as a potential genetic marker for growth traits. Studies suggest that SNPs in the *mstn* gene can influence growth performance (Sun et al., 2012; Wang et al., 2014; Nazari et al., 2016; Yang et al., 2018). In this study, we identified two SNPs (g.818 A>G, g.1003 T>C) in exon 2 of the hybrid grouper’s *mstn* gene at Hardy-Weinberg Equilibrium (HEW). Only one SNP (g.1003 T>C) displayed significant associations with six growth traits, with the TT genotype predominating over the two genotypes. Both non-coding and coding regions’ SNPs have been linked to the regulation of growth performance (Yu et al., 2007; Liu et al., 2012; Sun et al., 2012; Nazari et al., 2016). Sequence variants in the non-coding region could affect RNA cleavage, stability, translation, export, and intracellular localization, ultimately altering gene expression (Pesole et al., 2001; Antonarakis and Cooper, 2003). Variants in the coding region could modify amino acid composition, leading to changes in protein structure (Niu et al., 2015). In our results, the g.1003 T>C was synonymous, and did not result in an amino acid change. Synonymous SNPs are considered to be functionally meaning (Sauna et al., 2007; Kerna et al., 2013). It can influence secondary structures of mRNA and thereby alter the length of pause cycles during translation, the overall rate of translation, or protein folding (Kimchi-Sarfaty et al., 2007; Bartoszewski et al., 2010). This ubiquitous long pauses can result in translational frame shifting and to protein misfolding (Wen et al., 2008). Synonymous mutation might be associated with a growth-related quantitative trait loci (QTL), thus contributing to growth performance modulation (Liu et al., 2012; Sun et al., 2012). In conclusion, polymorphisms in the *mstn* gene could serve as molecular markers to improve the growth of hybrid grouper, facilitating marker-assisted selective breeding.

## 5 Conclusion

Growth is a crucial economic trait in farmed grouper. The *mstn* gene is a significant growth factor that negatively regulates skeletal muscle development and growth. Investigations into the correlation between growth traits, DNA methylation levels, and genetic variations can provide fundamental insights to enhance grouper growth performance. Consequently, this study examined the DNA methylation level in a hybrid grouper (*E*. *fuscoguttatus* (♀) × *E*. *polyphekadion* (♂)) and its parent species. Significant differences in DNA methylation sites were discovered between the hybrid grouper and *E. polyphekadion* at sites 824 and 1,521 (located at exon 2 and intron 2, respectively), and between *E. fuscoguttatus* and *E. polyphekadion* at site 1,521. These differences can potentially influence the mRNA expression of the *mstn* gene. Moreover, this study screened SNPs of the *mstn* gene associated with growth traits in the hybrid grouper. The results revealed an SNP at position g.1003T > C in the *mstn* gene. The TT genotype was found to be dominant at this site and demonstrated superior growth performance compared to individuals with the TC genotype. Histological examination via HE staining further corroborated these findings, showing that the average area and diameter of muscle fibers in individuals with the TT genotype were greater than those in individuals with the TC genotype.

## Data Availability

The datasets presented in this study can be found in online repositories. The names of the repository/repositories and accession number(s) can be found below: GenBank database (BioProject ID: PRJNA1009792).

## Supplementary material

SUPPLEMENTARY FIGURE S1 Graphical map of *mstn* nucleotide sequence in the hybrid grouper.

SUPPLEMENTARY FIGURE S2 Sequencing maps of SNPs of *mstn* gene in sites 818 **(A,B)** and 1003 **(C,D)**, respectively. All the sequences were reverse complementary sequences.
