# Supplementary figures and images for: Exploring the influence of DNA methylation and single nucleotide polymorphisms of the Myostatin gene on growth traits in the hybrid grouper (Epinephelus fuscoguttatus (female) × Epinephelus polyphekadion (male))
Source: Front Genet. 2024 Jan 8;14:1277647. doi: 10.3389/fgene.2023.1277647 (PMC10801740; doi:10.3389/fgene.2023.1277647)

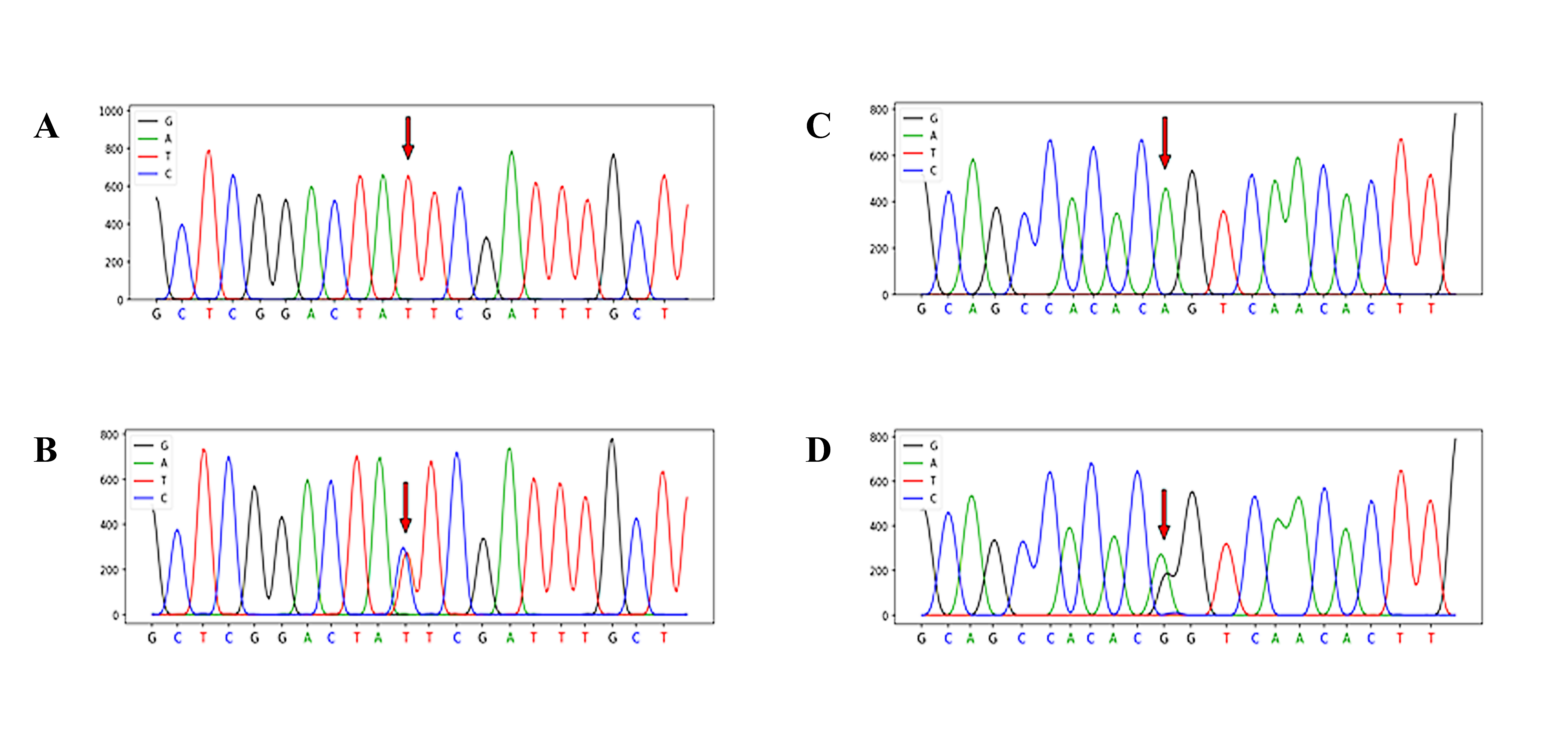

Supplement: Supplementary file 1 [file Image2.TIF]

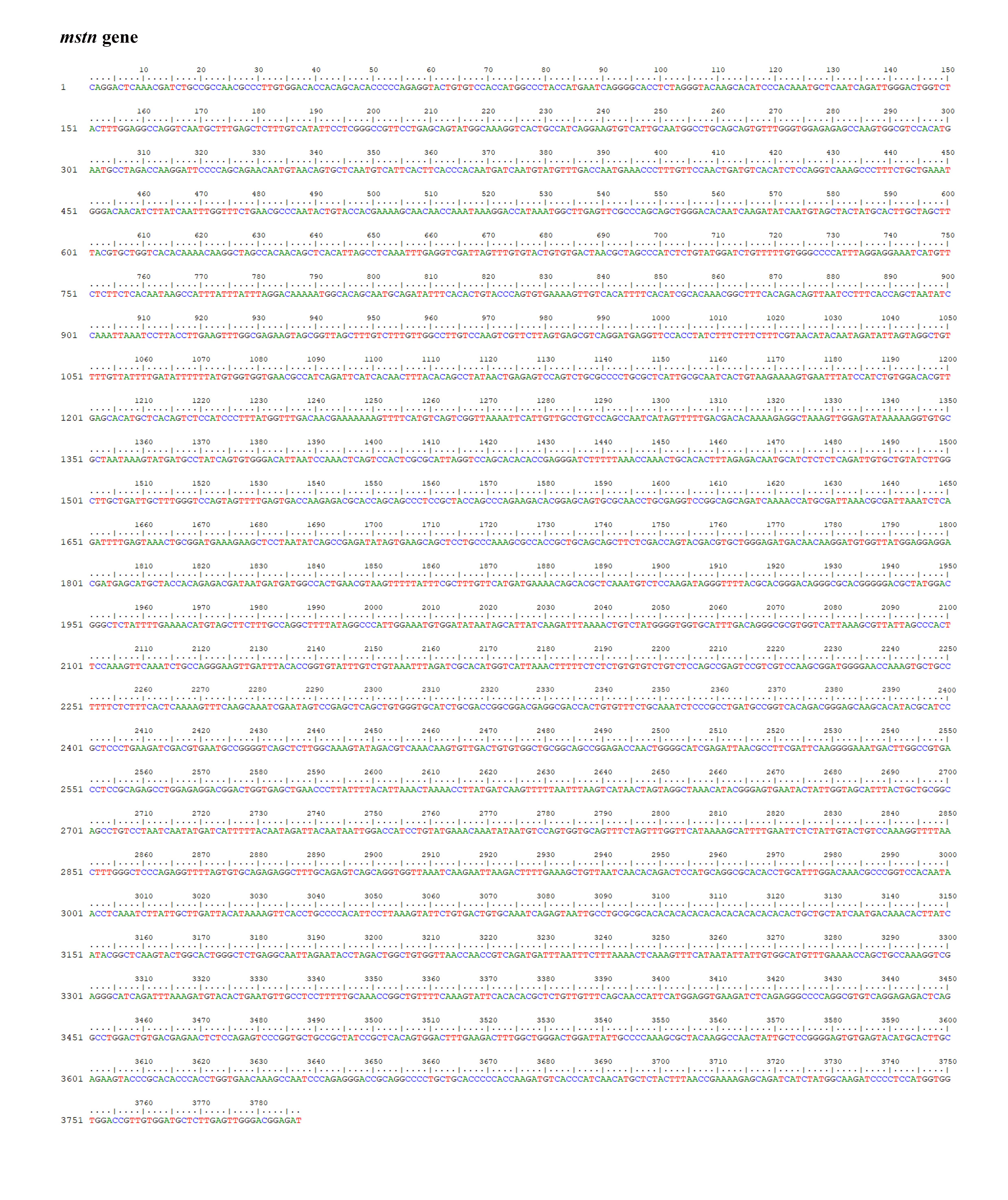

Supplement: Supplementary file 2 [file Image1.TIF]
