# Supplementary material for: Exploring the influence of DNA methylation and single nucleotide polymorphisms of the Myostatin gene on growth traits in the hybrid grouper (Epinephelus fuscoguttatus (female) × Epinephelus polyphekadion (male))
Source: Front Genet. 2024 Jan 8;14:1277647. doi: 10.3389/fgene.2023.1277647 (PMC10801740; doi:10.3389/fgene.2023.1277647)
Supplement: Supplementary file 3 [file Table1.DOC]

Table S1 The primers used for SNP-specific PCR and BSP-PCR

|  | Sequences (5’-3‘) | Usage |
| --- | --- | --- |
| Primer 1 | F: AACTGAGAGTCCAGTCTGCG | Searching SNP sites |
|  | R: AGTCATTTCCCCTTGAATCG |  |
| Primer 2 | F: GGAGCAAGCACATACGCATC | Searching SNP sites |
|  | R: TGAGCGGATAGCGGCAGCAC |  |
| Primer 3 | F: GTCGTCCAAGCGGATGGGGA | Verifying SNP site |
|  | R: AGTCATTTCCCCTTGAATCG |  |
| Primer 4 | F: TTTTGATATTTTTTATGTGGTGGTGAAYGTTATTAG | Bisulfite sequencing |
|  | R: CTATAATTAACTAAACAAACAACAATAAATTTTAACC |  |
| Primer 5 | F: GAAGATAYGGAGTAGTGYGTAATTTG | Bisulfite sequencing |
|  | R: ACATCCTTATTATCATCTCCCAACAC |  |
| Primer 6 | F: ATAGAGAYGATAATGATGATGGTTATTG | Bisulfite sequencing |
|  | R: CATTTCCAATAAACCTATAAAAACCTAAC |  |
| Primer 7 | F: GGATGGGGAATTAAAGTGTTGTTTTT | Bisulfite sequencing |
|  | R: TCTATACTTTACCAAAAACTAACCCC |  |
| Primer 8 | F: ATTTTTGAAAGTTGTTAATTAATATAGATTTTATGTAGG | Bisulfite sequencing |
|  | R: CAAAACCCAATACCAATACTTAAACC |  |
| Primer 9 | F: GTGCTGTATCTTGGCTTGCT | qPCR |
|  | R: ACTGCTCCGTGTCTTCTGG |  |
| β-actin | F: GAGAGGTTCCGTTGCCCAGAG | qPCR |
|  | R: CAGACAGCACAGTGTTGGCGT |  |
